# Maternal Exposure to Polycyclic Aromatic Hydrocarbons and 5’-CpG Methylation of Interferon-γ in Cord White Blood Cells

**DOI:** 10.1289/ehp.1103744

**Published:** 2012-05-04

**Authors:** Wan-yee Tang, Linda Levin, Glenn Talaska, Yuk Yin Cheung, Julie Herbstman, Deliang Tang, Rachel L Miller, Frederica Perera, Shuk-Mei Ho

**Affiliations:** 1 Division of Environmental Genetics and Molecular Toxicology,; 2 Center for Environmental Genetics,; 3 Division of Epidemiology and Biostatistics, and; 4 Division of Industrial Hygiene and Occupational Safety, Department of Environmental Health, College of Medicine, University of Cincinnati, Cincinnati, Ohio, USA; 5 Columbia Center for Children’s Environmental Health, Columbia University Mailman School of Public Health, New York, New York, USA; 6 Division of Pulmonary, Allergy and Critical Care Medicine, Columbia University College of Physicians and Surgeons, New York, New York, USA; 7 Cancer Institute, College of Medicine, University of Cincinnati, Cincinnati, Ohio, USA; 8 Cincinnati Veteran Affairs Medical Center, Cincinnati, Ohio, USA

**Keywords:** cord white blood cell, cytokines, DNA methylation, epigenetic epidemiology, epigenetics, fetal origins of disease, interferon-γ, interleukin 4

## Abstract

Background: Maternal factors are implicated in the onset of childhood asthma. Differentiation of naïve CD4^+^ T lymphocytes into pro-allergic T-helper 2 cells induces interleukin *(IL)4* expression and inhibits interferon *(IFN)*γ expression accompanied by concordant methylation changes in the promoters of these genes. However, it has yet to be established whether maternal exposure to polycyclic aromatic hydrocarbons (PAHs) can alter these gene promoters epigenetically during fetal development.

Objectives: In this study we sought to elucidate the relationship between maternal PAH exposure and promoter methylation status of *IFN*γ and *IL4*.

Methods: We assessed the effects of benzo[*a*]pyrene (BaP), a representative airborne PAH, on the methylation status of the *IFN*γ and *IL4* promoters in Jurkat cells and two lung adenocarcinoma cell lines, and on gene expression. In addition, we evaluated methylation status of the *IFN*γ promoter in cord white blood cells from 53 participants in the Columbia Center for Children’s Environmental Health cohort. Maternal PAH exposure was estimated by personal air monitoring during pregnancy.

Results: *In vitro* exposure of the cell models to low, noncytotoxic doses (0.1 and 1 nM) of BaP elicited increased promoter hypermethylation and reduced expression of *IFN*γ, but not *IL4*. *IFN*γ promoter methylation in cord white blood cells was associated with maternal PAH exposure in the cohort study subsample.

Conclusion: Consistent with the results for the cell lines, maternal exposure to PAHs was associated with hypermethylation of *IFN*γ in cord blood DNA from cohort children. These findings support a potential role of epigenetics in fetal reprogramming by PAH-induced environmental diseases.

The dysregulation of specific T lymphocytes and their cytokines plays an important role in the etiology of allergic asthma (Ray et al. 2010). The onset of allergic asthma is characterized by increased infiltration of naïve CD4^+^ T lymphocytes into the bronchial mucosa. Upon sensitization by allergens, activated dendritic cells initiate the differentiation of naïve CD4^+^ T cells into proallergic T-helper (Th) 2 cells instead of the counterregulatory Th1 cells (Hammad and Lambrecht 2006). The progressive increase in the commitment of CD4^+^ T cells toward a Th2 phenotype is accompanied by an upregulation of Th2 cytokines, such as interleukin (IL) 4, and the silencing of Th1 cytokines like interferon (IFN) γ (Ray et al. 2010).

Allergic asthma has been hypothesized to be a disorder of fetal origin that is influenced greatly by maternal factors (Ho 2010). However, molecular mechanisms underlying the potential effects of maternal exposures on asthma remain unclear. We are currently studying a cohort of African-American and Dominican children living in a traffic-laden area of Northern Manhattan and South Bronx, New York, where asthma prevalence greatly exceeds national rates (Centers for Disease Control and Prevention 2011). Prenatal exposure to traffic-derived pollutants, such as polycyclic aromatic hydrocarbons (PAHs), may account for some of the increased asthma prevalence in this cohort (Tonne et al. 2004). We previously analyzed DNA isolated from umbilical cord white blood cells (UCWBCs) in a subset of cohort children and identified six genes whose methylation status was associated with maternal PAH exposure using a methylation-sensitive restriction fingerprinting approach (Perera et al. 2009). Of these, acyl-CoA synthetase long-chain family member 3 (ACSL3) exhibited the highest concordance between the promoter methylation in UCWBC DNA and the level of gene expression in matched fetal placental tissues. *ACSL3*, a gene that encodes a key enzyme in fatty acid metabolism, was significantly associated with maternal PAH exposure and with reported childhood asthma through 5 years of age. These findings support the emerging theory of developmental reprogramming of later-life disease risk by exposure to epigenetically active environmental agents, and led us to hypothesize that genes known to be involved in the etiology of allergic asthma might be dysregulated in a similar manner.

Hence, our objective was to elucidate the relationship between maternal PAH exposure and the methylation status of two genes encoding Th1/Th2 regulatory cytokines—*IFN*γ and *IL4* (Ho 2010; Kuriakose and Miller 2010). Previous studies have identified specific CpG sites flanking the transcriptional start site (TSS) of *IFN*γ that regulate gene expression in human cord blood CD4^+^ T cells (White et al. 2002) and undergo demethylation when naïve CD4^+^ T cells differentiate into Th1 cells (White et al. 2006) or hypermethylation during Th2 polarization (Jones and Chen 2006). To our knowledge, no studies have addressed whether prenatal changes in the methylation status of 5´ CpG islands (CGIs) or CpG sites in *IFN*γ and *IL4* are associated with maternal PAH exposure.

Here, we evaluated the direct effects of benzo[*a*]pyrene (BaP), a representative PAH, on the methylation status of the 5´-flanking region and gene expression of *IFN*γ and *IL4* in Jurkat T cells and two adenocarcinoma cell lines (A549 and H1793) as *in vitro* models of epigenetic responses of immune or airway cells to PAH exposure. Next, we determined whether the degree of promoter methylation in *IFN*γ and/or *IL4* in UCWBCs from a subset of 53 newborns in the Columbia Center for Children’s Environmental Health (CCCEH) cohort (Perera et al. 2009) were correlated with maternal PAH exposure.

## Methods

*Treatment of cell lines with BaP.* Jurkat T cells were maintained in RPMI media (Invitrogen, Carlsbad, CA) supplemented with 10% fetal bovine serum (FBS). Two lung adenocarcinoma cell lines, A549 and H1793 were maintained in F12K (Invitrogen) and Dulbecco’s modified Eagle medium (DMEM)/F12, respectively. Both media were supplemented with 10% FBS (Hyclone, Logan, UT). For H1793 cultures, the DMEM/F12 medium was supplemented with 10 nM hydrocortisone, estradiol-17β (Sigma, St. Louis, MO), 1X insulin/transferrin/selenite, and 4.5 mM l-glutamine (Invitrogen). For BaP treatment, cells were exposed to 0.1, 1.0, 10, or 100 nM BaP (Sigma) or with DMSO alone as control, every other day for 4 days. Total RNA and DNA were isolated as previously described (Perera et al. 2009).

*Real-time reverse-transcriptase-PCR (RT-PCR).* Total RNA was reverse transcribed and the transcript levels quantified by SYBR Green-based RT-PCR as previously described (Perera et al. 2009). The following primers were used for the quantification of transcripts of β*-actin* (X00351.1): Fwd-5´-GC​GG​GA​AA​TC​GT​GC​GT​GA​CA​TT-3´ and Rev-5´-GA​TG​GA​GT​TG​AA​GG​TA​GT​TT​CG​TG-3´; *IFN*γ (NM_000619.2): Fwd-5´-TT​TG​GG​TT​CT​CT​TG​GC​TG​TT-3´ and Rev-5´-CT​GT​CA​CT​CT​CC​TC​TT​TC​CAA-3´); and *IL4* (NM_000589.2 and NM_172348.1): Fwd-5´-TG​AA​CA​GC​CT​CA​CA​GA​GC​AG-3´ and Rev-5´-CT​CT​GG​TT​GG​CT​TC​CT​TC​AC-3´. Mean transcript levels in cell cultures were obtained from at least three separate experiments. The 2-ΔΔCt method was used to calculate the relative expression levels of a transcript by normalization to the level of β-actin mRNA. Values in vehicle-treated (BaP: 0 nM) cells were assigned arbitrarily an abundance value of 1.00 for each gene and values from other treatment groups were compared with values in the vehicle-treated cultures.

**Figure 1:**
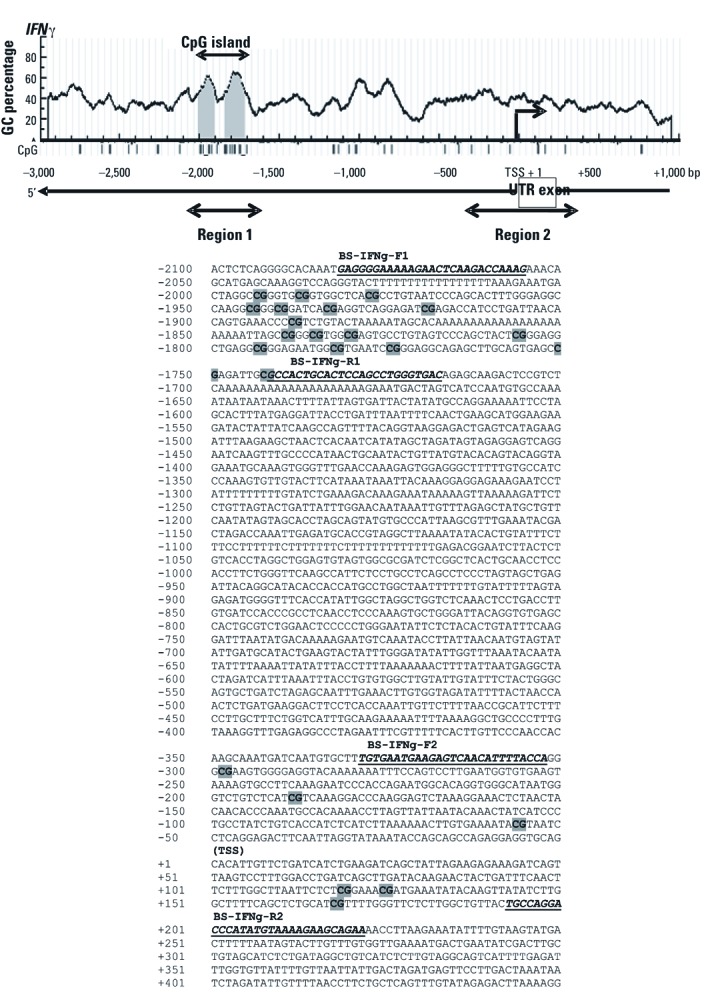
CG content (%) in the 5’ flanking region of *IFN*γ and the location of primers used in this study. The CGI(s) (shaded in gray) were identified *in silico* based on a CG content of 60%, with an observed:expected ratio of 0.6 according to instructions from MethPrimer. UTR exon: untranslated exon (shown in box). The PCR-amplified regions (regions 1 and 2) are indicated by double arrow heads; the methylation status of this region was determined by bisulfite genomic sequencing. The primers (BS–IFNg–F1, –R1, –F2, and –R2) for PCR are in dark italic bold type. There are 17 and 6 CG sites encompassing regions 1 and 2, respectively. An individual CG site is marked as gray in the genomic DNA sequence.

*Bisulfite genomic sequencing.* Bisulfite genomic sequencing was conducted according to previously described protocols (Perera et al. 2009). Genomic DNA was bisulfite-modified before PCR. *In silico* analyses were used to predict the 5´-CGI(s) of *IFN*γ (NC_000012:c66843688-66838788) (Figure 1) and *IL4* [NC_000005.9:132007373-132010373; see Supplemental Material, Figure S1 (http://dx.doi.org/10.1289/ehp.1103744)]. Primers were designed to amplify a 365 bp (–2082 to –1718) fragment upstream of TSS encompassing the predicted 5´-CGI(s) of *IFN*γ (Region 1: BS-IFNg-F1: 5´-GAGG​GG​AA​AA​AG​AA​TT​TA​AG​AT​TA​AAG-3´ and BS-IFNg-R1:5´-AT​CA​CC​CA​AA​CT​AA​AA​TA​CA​AT​AAC-3´) and a 551 bp (–329 to +222) fragment including 6 CpG dinucleotides flanking the TSS of the gene (Region 2: BS-IFNg-F2: 5´-TGTG​AA​TG​AA​GA​GT​TA​AT​AT​TT​TA​TTA-3´ and BS-IFNg-R2: 5´-TTCT​AC​TT​CT​TT​TA​CA​TA​TA​AA​TC​CT​AACA-3´) from bisulfite-modified DNA. Similarly, a pair of primers was used to amplify a 337 bp (–1388 to –1052) fragment of the predicted 5´-CGI of *IL4* (BS-IL4-F:5´-GGGA​AG​TG​GA​AT​AG​AG​GT​AA​AA​TTT-3´ and BS-IL4-R:5´-ATCA​CC​CA​AA​CT​AA​AA​TA​CA​AT​AAC-3´). Six clones were picked from individual UCWBC samples, and a total of 12 clones were picked from three individual sets of cell line samples and sequenced (Macrogen, Rockville, MD).

*Cell cytotoxicity assay.* Jurkat T cells, A549, and H1793 were treated with BaP (0, 0.1, 1, 10, or 100 nM), or with DMSO as control, every 2 days for a total of 4 days. Triplicate experiments were performed. Percentages of viable lung cancer cells following BaP exposure were determined by cell proliferation assay using MTS solution (Promega, Madison, WI). For Jurkat T cells, the numbers of viable cells present in treated or untreated samples were counted after staining cells with Tryptan Blue (Invitrogen).

*^32^P-Postlabeling analysis.* DNA adducts were measured according to published protocols (Talaska et al. 1990, 1991). A brief description of the procedures is provided in Supplemental Material, p. 3 (http://dx.doi.org/10.1289/ehp.1103744).

*Study population.* A total of 727 nonsmoking African-American and Dominican women and their children, who lived in a high-traffic area of Manhattan and South Bronx were enrolled in the CCCEH cohort study as previously described (Miller et al. 2004; Perera et al. 2003; Perzanowski et al. 2006). Written informed consent was obtained from all subjects following procedures approved by the Institutional Review Board of the New York–Presbyterian Medical Center. Completion of prenatal air monitoring for the mothers and collection of umbilical cord blood sample at delivery were required. We used the same study sample described previously (Perera et al. 2009). Briefly, 53 cohort participants were dichotomized on the median PAH levels of the CCCEH cohort (2.27 ng/m^3^), including 27 selected from those above the median (high PAH exposure) and 26 selected from those below the median (low exposure). This sample was representative of the cohort population regarding maternal age, child sex, median maternal PAH exposure, and percentage of children with probable asthma, but differed with respect to ethnicity (full cohort, *n* = 606, 63.5% Dominican and 36.5% African-American; study sample, *n* = 53, 49% Dominican and 51% African-American).

*Monitoring and sample collection.* Maternal PAH exposure was assessed from personal prenatal air monitoring during the third trimester of pregnancy as previously described (Perera et al. 2003). In brief, total PAH exposure levels for each mother were calculated as the sum of eight nonvolatile (molecular weight 228–278: benzo[*a*]anthracene, chrysene/iso-chrysene, benzo[*b*]fluoranthene, benzo[*k*]fluoranthene, BaP, indeno[1,2,3-*c,d*]pyrene, dibenzo[*a,h*]anthracene, benzo[*g,h,i*]perylene) carcinogenic PAHs collected as part of total dust on the filters of the personal monitors. Samples with total PAH < 0.125 ng/m^3^ were considered below the limit of detection. Umbilical cord blood (30–60 mL) was collected at delivery. The buffy coat, packed red blood cells, and plasma samples were separated and stored at –80°C. DNA (100–500 ng) was extracted from the buffy coat. All samples were de-identified.

*Statistical methods.* The distributions of characteristics of 53 participants were compared after stratification at the median level of maternal PAH exposure levels, 2.27 ng/m^3^. Pearson’s chi-square test of independence was used for comparing exposure levels with respect to ethnicity (percent African American vs. Dominican), sex (percent male), maternal exposure to tobacco smoke during pregnancy (ETS) (percent yes), and receipt of public assistance (percent yes). Differences between medians of age at delivery (years) were compared by quantile regression. Based on the median as an approximation to the center of subsets of distributions of percent methylation, quantile regressions were performed to estimate differences between the medians of percent methylation for the specified categories of each characteristic. We included receipt of public assistance as an indicator of family socioeconomic status (SES) because it was more complete (no missing data) and considered to be more objective than some of the other available measures of SES. The statistical analyses focused on estimating the association between maternal PAH exposure and DNA percent methylation using multiple regression analyses, adjusted for participant characteristics. Because of the skewness of the distribution of PAH, the log_e_-transformation (ln-PAH) was applied to improve symmetry.

Generalized additive models (GAMs) were analyzed in which smooth plots of region-specific curves relating percent methylation of the *IFN*γ promoter to ln-PAH and age at delivery were drawn. General regressions were analyzed where ln-PAH and age at delivery were modeled as restricted cubic spline functions with turning points (knots), identified by the GAMs. A (restricted) cubic spline function consists of piecewise cubic polynomial functions relating a dependent and independent variable. The parameters of the polynomial functions depend on the number and location of specified knots. In addition to an intercept term, the number of parameters required is one less than the number of knots, because the polynomials are constrained to be linear in the tails. Thus, a restricted cubic spline function with three knots will require two parameters. The first parameter describes the effect of the linear component of the independent variable; the second parameter describes the effect of the nonlinear component. The linear and nonlinear components of the function should not be separated in interpreting the trend that is graphically displayed.

Regressions were adjusted for participant characteristics, ethnicity, sex, ETS exposure, and receipt of public assistance as dichotomous variables modeled dichotomously, and log_e_-percent (ln-percent) methylation was the dependent variable (Harrell 2001). This transformation improved the fit of the full and reduced models, compared with the untransformed analyses, of each region, as determined by the Aikaike Information Criterion. Restricted cubic splines for ln-PAH were constructed with knots at the 10th and 90th percentiles and a third knot at 3.5 ng/m^3^ (ln-PAH = 1.3). Splines for age at delivery varied according to IFNγ region (at the 10th and 90th percentiles and at third knot at 25 for region 1; at the 5th and 95th percentiles for region 2.) Reduced models included covariates that predicted the outcome with *p* < 0.20. The significance of the linear and nonlinear terms of the restricted cubic spline functions were evaluated by the likelihood ratio test statistic (LRT), where subsequent models were reduced by testing nonlinear terms of the spline function first. Spline terms were retained if *p* < 0.20. The analyses were performed using SAS for Windows, version 9.2 (SAS Institute Inc., Cary, NC). Graphs were generated using S-Plus software, S-Plus 2000 (TIBCO Software Inc., Palo Alto, CA). An alpha level of 0.05 indicates statistical significance.

## Results

IFNγ *promoter methylation and transcription. In silico* analysis of the *IFN*γ 5´ flanking region revealed a 287 bp CGI (–2061 to –1717) upstream of the TSS (Figure 1). In addition, several CpG sites (distal to the CGI) within the region between –329 to +220, flanking the TSS of *IFN*γ, were previously reported to be aberrantly methylated during Th1/Th2 differentiation and T cell stimulation (Janson et al. 2008; Liu et al. 2008). Therefore, we performed methylation studies on both CGI (region 1) and non-CGI CpG dinucleotides (region 2).

**Figure 2:**
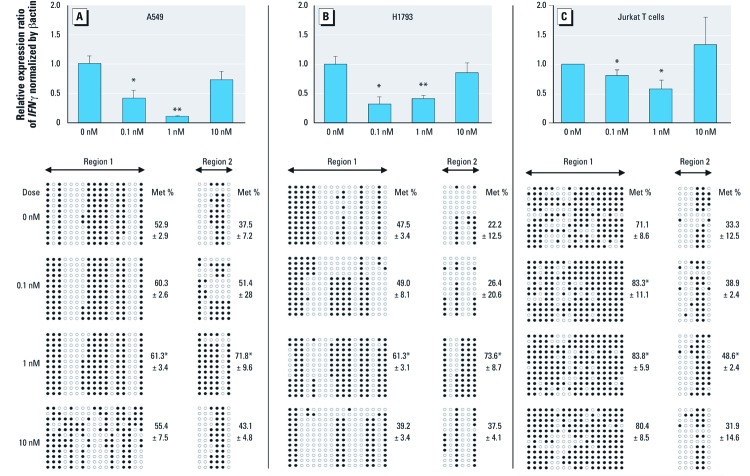
Real-time RT-PCR analysis of *IFN*γ gene expression (upper panel) and bisulfite genomic sequencing analysis of *IFN*γ promoter methylation status (lower panel) in lung cancer A549 cells (*A*), H1793 cells (*B*) and Jurkat T cells (*C*) in response to BaP. Cells were treated with 0.1, 1, or 10 nM BaP, with DMSO as control, every 2 days for a total of 4 days. Triplicate experiments were performed. RNA was isolated, reverse transcribed, and amplified by RT-PCR. The 2-ΔΔCt method was used to calculate the relative expression level of *IFN*γ transcripts normalized to β-actin. Genomic DNA was isolated and bisulfite treated prior PCR amplification of promoter regions 1 and 2. PCR products were subcloned into pCR2.1 vectors. Four individual clones from each experiment and a total of 12 clones from each BaP concentration were sequenced. Each row represents an individual clone of the promoter. A total of 17 CpG sites on region 1 and 6 CpG sites on region 2 were analyzed. Each circle represents a CpG site within the promoter: Open circles represent unmethylated CpGs and closed circles represent methylated CpGs. Met %, average percent of total CpG methylation. Error bars are SDs obtained from triplicate experiments. *p < 0.05 or **p < 0.01 compared with untreated controls.

We first investigated whether gene silencing was accompanied by changes in the methylation status of region 1 and/or region 2 of the *IFN*γ 5´ flanking sequence (Figure 2). Here, we used the two pulmonary epithelial carcinoma cell lines derived from a male (A549) and a female (H1793) patient and the Jurkat T cells, an immortalized line of T lymphocytes derived from a male patient with T-cell leukemia (Gillis and Watson 1980), to determine whether BaP induces *IFN*γ expression and promoter methylation in these cell lines representing pulmonary and immune epithelial cell models. *IFN*γ expression was significantly lower than in controls following exposure to 0.1 and 1 nM BaP in all 3 cell lines (*p* < 0.05). However, *IFN*γ expression was not significantly different from controls after exposure to a higher dose of BaP (10 nM) (Figure 2). Decreased *IFN*γ expression was associated with enhanced promoter methylation induced by 1 nM of BaP at regions 1 and 2 for all three cell lines (Figure 2). In concordance with gene expression data, a higher dose of BaP (10 nM) was not significantly associated with *IFN*γ promoter methylation in any cell line. The lack of significant effects of 10 nM BaP on DNA methylation and gene expression may have reflected cytotoxicity [see Supplemental Material, Figure S2 (http://dx.doi.org/10.1289/ehp.1103744)]. Specifically, decreased cell viability was observed in both lung cell lines treated with 10 or 100 nM of BaP, and in Jurkat T cells exposed to 1, 10, or 100 nM of BaP. BaP–DNA adduct formation was increased in both lung cell lines following treatment, but levels were significantly different from controls following 1 or 100 nM BaP in H1793 cells and 100 nM BaP in A549 cells (see Supplemental Material, Figure S3).

IL4 *methylation and transcription.* We assessed the methylation status of the *IL4* gene 5´ flanking region and its expression by bisulfite genomic sequencing and RT-PCR in Jurkat T cells, A549, and H1793. *IL4* expression was decreased significantly in A549 cells exposed to low concentrations (0.1 and 1 nM) of BaP, and significantly increased in BaP-treated H1793 cells (0.1 and 1.0 nM) and Jurkat T cells (1.0 nM) (*p* < 0.05) [see Supplemental Material, Figure S4 (http://dx.doi.org/10.1289/ehp.1103744)]. However, there was no significant difference in promoter methylation in cells exposed to BaP compared with controls. Therefore, *IL4* was excluded from methylation studies on the UCWBC samples.

*Maternal PAH exposure and* IFNγ *promoter methylation in cohort samples.* Next, we determined whether there were differences in *IFN*γ promoter methylation between UCWBC DNA samples and their corresponding maternal PAH exposures. Participant characteristics [see Supplemental Material, Table S1 (http://dx.doi.org/10.1289/ehp.1103744)] were similar (*p* > 0.05) when stratified by maternal PAH exposure (< 2.27 or ≥ 2.27 ng/m^3^). Median percent methylation in *IFN*γ region 1 was significantly higher among participants with high versus low PAH exposure (97.1% vs. 88.7%, *p* < 0.01) (see Supplemental Material, Table S2). Median percent methylation in *IFN*γ region 2 was significantly higher among Dominicans than among African Americans (96.7% vs. 92.5%, *p* = 0.04), and higher, with borderline significance (*p* = 0.06) for recipients of public assistance (95.8%) compared with nonrecipients (91.7%). There were no other results that approached significance with respect to differences in median percent methylation for dichotomized participant characteristics.

**Figure 3:**
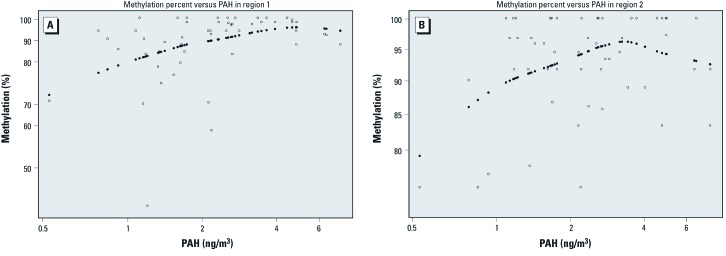
Smoothed plots relating predicted values of percent methylation of regions 1 (*A*) and 2 (*B*) to PAH levels for 53 children. Predicted values were adjusted for participant characteristics shown in Supplemental Material, Table S3 (http://dx.doi.org/10.1289/ehp.1103744) final models. Open (closed) circles represent the observed (predicted) values of percent methylation for each child.

Linearly modeled maternal ln-PAH was significantly related to ln-percent methylation in the final model for *IFN*γ region 1 adjusted for sex and spline age at delivery [Figure 3A; also see Supplemental Material, Table S3 (http://dx.doi.org/10.1289/ehp.1103744)]. Based on the final model for *IFN*γ region 2, the relation between maternal ln-PAH and ln-percent methylation was significant when modeled as a restricted cubic spline function, adjusted for ETS, public assistance, and spline age at delivery. Predicted values of percent methylation increased up to approximately 3.5 ng/m^3^ PAH, after which they decreased (Figure 3B; also see Supplemental Material, Table S4). The relation between age at delivery and ln-percent methylation was modeled as a restricted cubic spline function in each region, as the LRT statistic was significant for adding the spline terms. Percent methylation for region 2 was significantly lower for recipients of public assistance than nonrecipients (*p* < 0.01). No other model covariates were statistically significant at the 5% level for either region.

## Discussion

Epigenetic alterations of genes driven by environmental exposure *in utero* or in early life have been associated with a variety of disorders and diseases during childhood and through adulthood (Ho 2010). These alterations frequently involve fluctuation in the methylation status of gene promoters accompanied by changes in gene expression. Allergic asthma is one of such afflictions that is being recognized increasingly as a disorder of fetal origin where maternal prenatal exposures appear to play a key role (Ho 2010). Prenatal exposure to PAHs has been associated with low birth weight and preterm birth, and these conditions were correlated with declined lung function in adults (Barker et al. 1991; Edwards et al. 2003; Perera et al. 2003). Our group previously reported that in cord blood samples methylation of a CGI in *ACSL3*, a gene involved in fatty acid metabolism, was associated with maternal exposure to PAHs and with reported asthma in children through 5 years of age (Perera et al. 2009). Interestingly, *ACSL3* is located in close proximity to 2q36, an asthma susceptibility locus found in Hutterite (Ober et al. 2000) and Puerto Rican (Choudhry et al. 2008) populations. Our present findings indicate that maternal exposure to PAHs is associated with the DNA methylation status of a known asthma gene, *IFN*γ.

In asthmatic patients, allergens induce the differentiation of naïve Th cells into pro-allergic Th2 cells accompanied by increased expression of Th2 cytokines such as IL4, and decreased expression of Th1 cytokines such as *IFN*γ in these cells for the allergic response (Ho 2010). Variations in *IL4* and *IFN*γ promoter methylation have been associated *in vitro* with polarized Th2 phenotypes, and has been demonstrated *in vivo* in Th cells of mice upon combined exposure to inhaled diesel exhaust particles (DEP) (a source of PAHs) and intranasal sensitization to *Aspergillus fumigatus* (Liu et al. 2008).

We hypothesized that maternal exposure to airborne PAH may induce altered methylation status of the asthma genes *IL4* and *IFN*γ. We used samples and data from participants in the CCCEH cohort who were included in our earlier study of *ACSL3* (Perera et al. 2009). Interestingly, *in vitro* exposure to BaP, a prototype PAH, elicited hypermethylation of the *IFN*γ promoter and decreased *IFN*γ expression in Jurkat T cells and two lung cancer cell lines. In contrast, changes in *IL4* gene expression were not associated with altered DNA methylation. This observation is at odds with a previous report of hypomethylation at CpG (–408) of the *IL4* promoter induced by combined exposure to inhaled DEP and intranasal *A. fumigatus* in mice splenic CD4^+^ cells (Liu et al. 2008). Our results also differ from those of Kwon et al. (2008), who reported demethylation of CpG (–80) in the *IL4* promoter of human CD4^+^ T lymphocytes isolated from patients with allergic asthma following *in vitro* exposure to dust mite allergens. A possible explanation is that synergism between PAH and an allergen may be necessary to alter the methylation status of the *IL4* promoter, such that BaP alone may not be sufficient to trigger IL4 promoter demethylation. In addition, changes in the methylation status of mouse CD4^+^ T cells may not extrapolate to other cell types or across species, or result from other environmental exposures.

Exposure of Jurkat T cells and lung cancer cell lines to BaP resulted in hypermethylation of a CGI in region 1 and specific non-CGI CpG sites in region 2 of the *IFN*γ promoter and reduced *IFN*γ expression, but these changes were significant only in cells treated with low doses (0.1 and 1 nM) of BaP. These apparent inverted U- or U-shape responses may reflect cytotoxicity in response to higher doses of BaP, consistent with our observation that cell viability decreased. On the other hand, DNA-adduct formation increased when cells were exposed to higher dose of BaP (100 nM). We speculated it may interfere with DNA methylation at the promoters although the relationship between DNA–adduct formation and DNA methylation has not been fully established. A recent paper demonstrated a significant positive correlation between PAH–adduct formation and hypermethylation of the promoter region of a tumor suppressor gene *TP53* in coke-oven workers (Pavanello et al. 2009). Further investigations are needed to unveil the mechanisms underlying this association.

*IFN*γ promoter methylation (in both regions 1 and 2) also was associated with maternal exposure to PAH in UCWBCs from children born to exposed women after adjusting for potential confounding factors. Our findings are the first to implicate hypermethylation of a CGI (region 1) located approximately 2 kb upstream of the TSS of the *IFN*γ promoter under the influence of maternal PAH exposure. Our results also confirm the role of methylation of non-CGI CpG sites flanking the TSS (region 2) in response to environmental pollutants, which has been reported in prior studies. Taken together, these findings suggest that a change in methylation status in either or both regions (the CGI and the non-CGI CpG sites flanking the TSS) of the *IFN*γ promoter sequence may be important for detecting environmental exposures to allergens/pollutants. The importance of CGI in gene regulation is well established (Ho 2010), but the significance of specific non-CGI CpG sites in gene regulation warrants further investigation.

Decreased expression of *IFN*γ via DNA methylation may promote differentiation of naïve cord blood Th cell repertoire into pro-allergic Th2 cells, but our study was not designed to address this mechanism specifically. In addition, other Th2 cytokines besides *IL4*, such as *IL5* and *IL13* that have been shown to be regulated by epigenetic mechanisms (Ho 2010; Webster et al. 2007) may be involved in the PAH effects. Interestingly, T regulatory cells, suppressor of allergic immune responses, were shown to be impaired by ambient air pollution (i.e., PAH) exposure and associated with increased *Foxp3* promoter methylation and worsened asthma severity scores of 71 children who participated in the Fresno Asthmatic Children’s Environment Study (Nadeau et al. 2010). Hence, it may be important in future studies to perform a detailed analysis of a more comprehensive panel of cytokines and their effects on transcription factor activation, to elucidate whether the absence of certain signaling components can be compensated by the overexpression of others. We also acknowledge that the present study involved small numbers of newborns from a selected population, thus limiting generalizability of these results. Last, we recognize the limitation of using cell lines (i.e., Jurkat T-cell leukemia, and A549 and H1793 lung adenocarcinoma cell lines) as *in vitro* models for elucidating the epigenetic responses of immune or airway cells to PAH exposure. Although we are aware that *in vitro* models cannot fully represent a complex disease such as asthma, each model can provide useful information concerning certain aspects of the disease.

In conclusion, we observed an association between maternal PAH exposure and promoter methylation of an asthma-related gene, *IFN*γ, in cord white blood cells from 53 children in our study cohort. In contrast, we have not observed any epigenetics at work modulating promoter methylation in *IL4*, in a manner related to maternal PAH exposure in this pilot. *IFN*γ, alone or in combination with other genes such as *ACSL3*, may be used as an epigenetic biomarker for environmental exposure to PAH if it can be validated in other birth cohort studies. Use of epigenetic biomarkers may facilitate the development of preventive measures for PAH-associated childhood asthma in the future.

## Supplemental Material

(348 KB) PDF
