# Supplementary material for: Maternal Exposure to Polycyclic Aromatic Hydrocarbons and 5’-CpG Methylation of Interferon-γ in Cord White Blood Cells
Source: Environ Health Perspect. 2012 May 4;120(8):1195–200. doi: 10.1289/ehp.1103744 (PMC3440069; doi:10.1289/ehp.1103744)
Supplement: (348 KB) PDF [file ehp.1103744.s001.pdf]

Supplemental Material

Maternal Exposure to Polycyclic Aromatic Hydrocarbons and 5'-CpG Methylation of Interferon-γ in Cord White Blood Cells

Wan-yee Tang[1,2,†], Linda Levin[2,3],Glenn Talaska[2,4], Yuk Yin Cheung[1], Julie Herbstman[5], Deliang Tang[5,] Rachel L Miller[5,6], Frederica Perera[5] and Shuk-Mei Ho[1,2,7,8*]

[1]Division of Environmental Genetics and Molecular Toxicology, Department of Environmental Health, College of Medicine, University of Cincinnati, Cincinnati, Ohio

[2]Center for Environmental Genetics, Department of Environmental Health, College of Medicine, University of Cincinnati, Cincinnati, Ohio

[3]Division of Epidemiology and Biostatistics, Department of Environmental Health, College of Medicine, University of Cincinnati, Cincinnati, Ohio

[4]Division of Industrial Hygiene and Occupational Safety, Department of Environmental Health, College of Medicine, University of Cincinnati, Cincinnati, Ohio

[5]The Columbia Center for Children's Environmental Health, Columbia University Mailman School of Public Health, New York City, New York

[6]Division of Pulmonary, Allergy and Critical Care Medicine, Columbia University College of Physicians and Surgeons, New York City, New York

[7]Cancer Institute, College of Medicine, University of Cincinnati, Cincinnati, Ohio

[8]Cincinnati Veteran Affairs Medical Center, Cincinnati, Ohio

[†]Current address: Division of Molecular and Translational Toxicology, Department of Environmental Health Sciences, Johns Hopkins Bloomberg School of Public Health, Baltimore, Maryland.

*Corresponding author: Shuk-mei Ho, Department of Environmental Health, Kettering Complex, Room 128, 3223 Eden Avenue, University of Cincinnati Medical Center, P.O. Box 670056, Cincinnati, OH 45267-0056; Tel: 513-558-5701; Fax: 513-558-0071; E-mail address: shuk-mei.ho@uc.edu

Table of Content

1. **Title Page** p.1

2. **Table of Content** p.2

3. **Supplemental Material, Methods**

3.1 ^{32}P-Postlabeling Analysis p.3

4. **Supplemental Material, Figures**

Figure S1 p.4-5

Figure S2 p.6

Figure S3 p.7

Figure S4 p.8

Table S1 p.9

Table S2 p.10

Table S3 p.11

Table S4 p.12

5. **Supplemental Material, Reference** p.13

Supplemental Material, Methods

^{32}P-Postlabeling Analysis

DNA adducts were measured according to published protocols. In brief, DNA was hydrolyzed to 3'-phosphodeoxynucleotides by incubation with 0.25 U micrococcal endonuclease and 2.5 μg calf spleen phosphodiesterase for 3 hours at 37°C. Nuclease P_1 was added to the digests to enhance the sensitivity of ^{32}P-postlabeling of PAH adducts (Reddy & Randerath 1986). Each sample was labeled with 50 μCi [^{32}P]-ATP in the presence of T4 polynucleotide kinase (U.S. Biochemical, OH). An aliquot (5-20 μl) of each sample was subjected to two-dimensional chromatography using polyethyleneimine cellulose-coated plastic plates, exposed to film, and the resultant autoradiogram was used to guide the excision of radioactive spots on the plates, as well as to excise a similar area to control for background activity. The total adducts were determined by summing the activities of adducts that appeared in the excised area. BaP-DNA adduct levels were expressed in RAL (Zavon M et al., 1973), which was defined as (cpm_{adduct} /$cpm_{unadducted\ nucleotide}$) x 10^9. All samples were analyzed in at least twice in separate experiments.

Supplementary Material, Figures

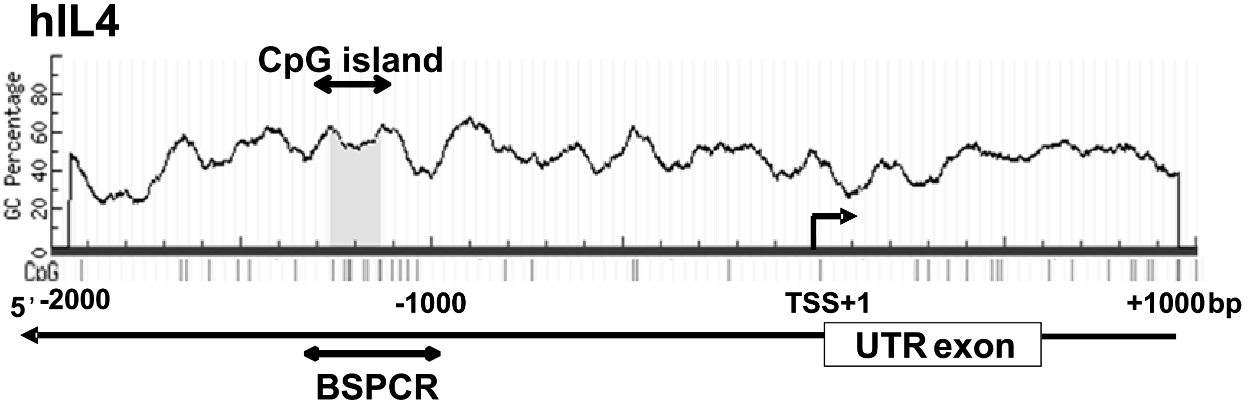


```
-2000   AGAATTGCTTGAGCTCAGGAGGCAGAGGTTGCAGTGAGCTGAGACTGCGC
-1950   CATTGCACTCCAGTCTGGGCAACAGAGCAAGACTCTATCTCAAAATAAAT
-1900   AAATAAATCACAGAGTAGATTTTAAATGTTCTTACCAAAAATAAATATGT
-1850   GAAGTATTGTATAAGTAGCTTGATGTAGCAATTCCATAACATGCACATTT
-1800   CAAAACATTATATCATACAGCACAAATATGTGCAATACTTATTTGTCAAT
-1750   TTAATAATAATAATAATAAGGGAAGAAAAGATCCAAAACAGAGGCAAAAC
-1700   CTTGGCCGGGCATGGTGGTTCACGCCTATAATCCCAGCACTTTGGGAGGC
-1650   TGAGGTGGGTGGATCATTTTGAGGCCAGGAGTTCGACACCAGCCTAGCCA
-1600   ACATGGTGAAACCCCATCTCTACTAAAAAAAAAAAAAAAAATACAGAAATT
-1550   AGCCAGGCGTGGTGGCATGTGCCTGTAATCCCAGCTACTCGGGAAGCTGA
-1500   GGCTGGAGAATGCCTTGAGCCCAGGAGATCAAGGCTACAGTAAGCTATGA
-1450   TCACCACTGCACTCCAGCCTGGGTGACAGAGTATGGGGGCAGGGGGTGGT
                    BS-IL4-F
-1400   GAGGGGGGGGCGGGGAAGTGGAACAGAGGCAAAACCTTAGCAACACACAT
-1350   TTTTAGATGATCTTCCAGAATAAATTCATAGGGAGGCCCAGGCACAGTGG
-1300   CTCACGCCTGTAATCCCAGCACTTTGGGAGGCCGAGGCAGGCGGATCACG
-1250   AGGTCAGGAGATGGAGACCATCCTGGCTAACACGGTGAAACCCCGTCTCT
-1200   ACTAAAAATACAAAAAATTAGCCGGGCGTGGTGGCAGGTGCCTGTAGTCC
-1150   CAGCTACTCGGGGAGGCTGAGGCAGGAGAACGGCATGAACCCAGGAGGCG
                                BS-IL4-R
-1100   GAGCTTGCAGTGAACTAAGATCACGCCACTGCACTCCAGCCTGGGTGACA
-1050   GAGCAAGATTCCATCTCAAAAAAAAAAAAAAAAAAAAAAGAAATTCATAGGG
-1000   AAAAGAAGGTCAGAGACCAAGGGAAGGGAAGGTTCTGGGAGAAAAGCAGG
-950    GGGCAGGCAGGGCCCAAGAATCCTGCTGCCCATGAGCCCTTACTGGGAGG
-900    TGGGGTGGCCTTGCACAGGGCCCAGGCACCTGAGTGAGTGGTGGGGTCCT
-850    TACGTTCACTGCTGGGGTGAGGCAATGAGCACCTTATTGTGTCCACATGA
-800    ATTCAATAAAAAACAAGCAGGGCGGGTGGTGGGGCACTGACTAGGAGGGC
-750    TGATTTGTAAGTTGGTAAGACTGTAGCTCTTTTTCCTAATTAGCTGAGGA
-700    TGTGTTTAGGTTCCATTCAAAAAGTGGGCATTCCTGGCCAGGCATGGTGG
-650    CTCACACCTGTAATCTCAGAGCTTTGGGAGACTGAGGTAGGAGGATCACT
-600    TGAGCCCAGGAATTTGAGATGAGCCTAGGCAACATAGTGAGACTCTTATC
-550    TCTATCAAAAAATAAAAATAAAAATGAGCCAGGCATGGTGCGGTGGCACG
-500    CACCTACTGCTAGGGGGGCTGAGGTGGGAGGATCACTTGAGCCTGGGAGG
-450    TTGAGGCTGCAGTGATCCCTGATCACAACATTGCATTTCAGCCTGGGTGA
-400    CAGAGTGAGACCCTGTCTCAGAAAAAAAAAAAAAAAAAGTCATTCCTGAAA
-350    CCTCAGAATAGACCTACCTTGCCAAGGGCTTCCTTATGGGTAAGGACCTT
-300    ATGGACCTGCTGGGACCCAAACTAGGCCTCACCTGATACGACCTGTCCTT
-250    CTCAAAACACCTAAACTTGGGAGAACATTGTCCCCCAGTGCTGGGGTAGG
-200    AGAGTCTGCCTGTTATTCTGCCTCTATGCAGAGAAGGAGCCCCAGATCAG
-150    CTTTTCCATGACAGGACAGTTTCCAAGATGCCACCTGTACTTGGAAGAAG
-100    CCAGGTTAAAATACTTTTCAAGTAAAACTTTCTTGATATTACTCTATCTT
-50     TCCCCAGGAGGACTGCATTACAACAAATTCGGACACCTGTGGCCTCTCCC
      (TSS)
+1      TTCTATGCAAAGCAAAAAGCCAGCAGCAGCCCCAAGCTGATAAGATTAAT
+51     CTAAAGAGCAAATTATGGTGTAATTTCCTATGCTGAAACTTTGTAGTTAA
+101    TTTTTTAAAAAGGTTTCATTTTCCTATTGGTCTGATTTCACAGGAACATT
+151    TTACCTGTTTGTGAGGCATTTTTTCTCCTGGAAGAGAGGTGCTGATTGGC
+201    CCCAAGTGACTGACAATCTGGTGTAACGAAAATTTCCAATGTAAACTCAT
+251    TTTCCCTCGGTTTCAGCAATTTTAAATCTATATATAGAGATATCTTTGTC
+301    AGCATTGCATCGTTAGCTTCTCCTGATAAACTAATTGCCTCACATTGTCA
+351    CTGCAAATCGACACCTATTAATGGGTCTCACCTCCCAACTGCTTCCCCCT
+401    CTGTTCTTCCTGCTAGCATGTGCCGGCAACTTTGTCCACGGACACAAGTG
+451    CGATATCACCTTACAGGAGATCATCAAAACTTTGAACAGCCTCACAGAGC
+501    AGAAGGTGAGTACCTATCTGGCACCATCTCTCCAGATGTTCTGGTGATGC
+551    TCTCAGTATTTCTAGGCATGAAAACGTTAACAGCTGCTAGAGAAGTTGGA
+601    ACTGGTGGTTGGTGGCAGTCCAGGGCACACAGCGAGGCTTCTCCCCTGCC
+651    ACTCTTTTTTCTGAGGGTTTGTAGGAAGTTTCCTCAGTTGGAGGGAGTGA
+701    GAGCTGCTCATCAAGGACTTCTCTGTCCGGTTGGAGGTTAACTCTGTCTC
+751    TTGCTCTCTCATTTCTGCCTGGACCAAGACTCTGTGCACCGAGTTGACCG
+801    TAACAGACATCTTTGCTGCCTCCAAGGTAAGAAGCCGTCCCACGGTCTGT
+851    TTTAGCAAATGGGGAGATCCATCCCCAAATGTCTGAACAAGAAACTTGTC
+901    TAATGGAAAACGAGCGGGCCCAAATTAACTCTAAGGTGTTAGATGTTTTC
+951    AAAGAACGAGAAGTCTGATCTTTACTCTTAAGCATGTTTTGGTCTTTCTG
```

Figure S1. Schematic diagram of CG content (%) in the 5' flanking region of *IL4*. The CGI(s) (shaded in gray) were identified *in silico* based on a CG content of 60%, with an observed/expected ratio of 0.6 according to instructions from MethPrimer. TSS: Transcription start site. UTR exon: untranslated exon The PCR-amplified regions were indicated by a line with double arrow heads and methylation status of this region was determined by bisulfite genomic sequencing. The primers (BS-IL4-F1 and -R1) for PCR are in dark italic bold type. There are 12 CG sites encompassing the entire CpG island and an individual CG site is marked as gray in the genomic DNA sequence.

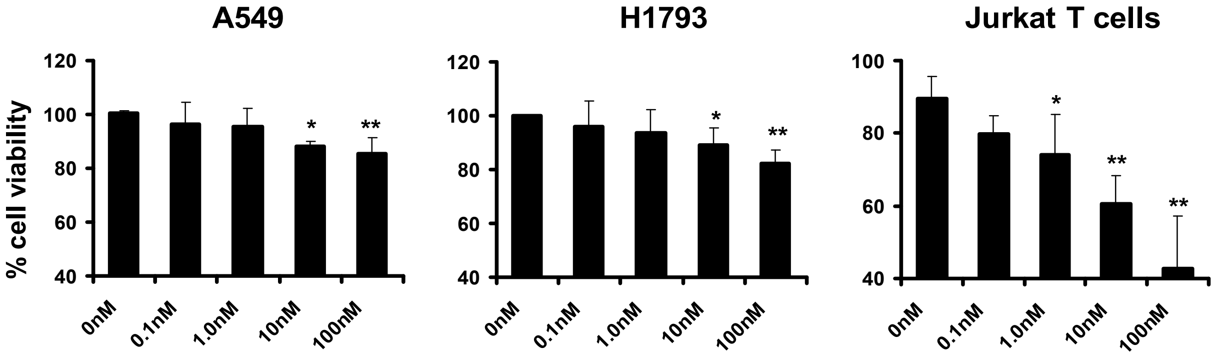

Figure S2. Cell Viability in exposure to BaP. Both lung cancer cells and Jurkat T cells were treated with 0.1, 1, 10 or 100nM BaP, with DMSO as control, every 2 days for a total of 4 days. Triplicate experiments were performed. Percentage of viable lung cancer cells in response to BaP were determined by cell proliferation assay using MTS solution. Cell viability of Jurkat T cells were measured by Tryptan Blue uptake and cell counting. * $p<0.05$ or ** $p<0.01$ compared with untreated control.

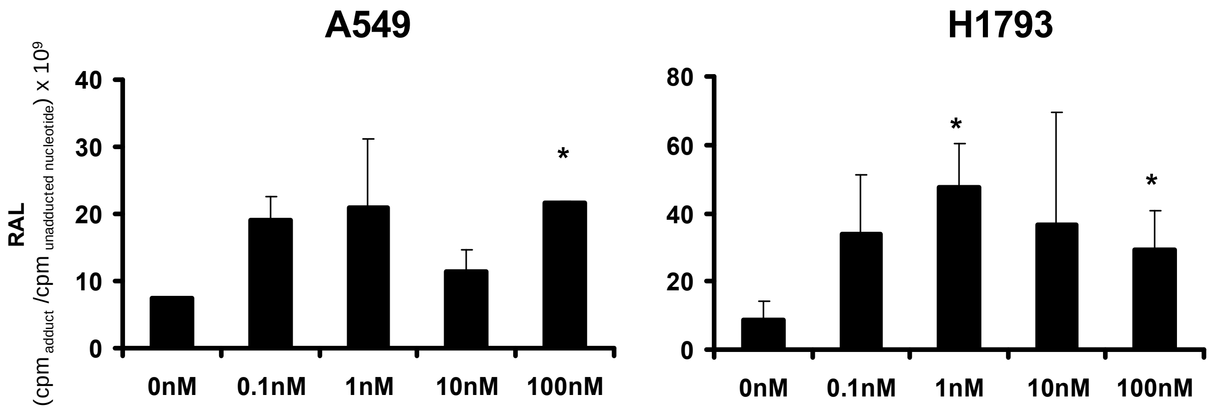

Figure S3. ^{32}P-Postlabeling analysis of BaP-DNA adduct formation. Lung cancer cells were treated with 0.1, 1, 10 or 100nM BaP, with DMSO as control, every 2 days for a total of 4 days. Triplicate experiments were performed. Genomic DNA was isolated, hydrolyzed and labeled with 50 µCi [^{32}P]-ATP. Sample was subjected to two-dimensional chromatography and exposed to X-ray film. The total adducts were determined by summing the activities of adducts that appeared in the area. BaP-DNA adduct levels were calculated by determining relative adduct labeling (RAL). All samples were analyzed in at least twice in separate experiments. Standard deviation was obtained from triplicate experiments and presented as error bar. * $p<0.05$ compared with untreated controls.

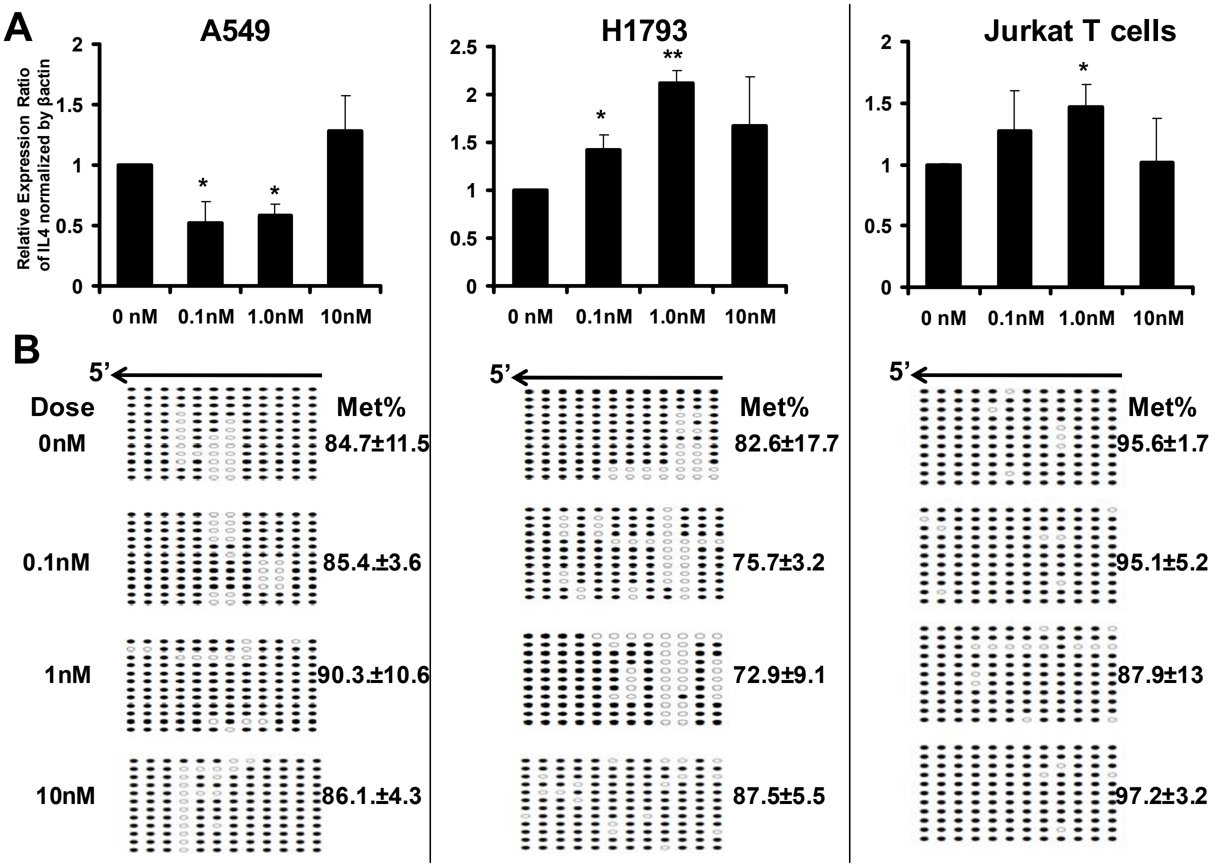

Figure S4. Real-time PCR analysis of gene expression of *IL4* (A) and bisulfite genomic sequencing analysis of the *IL4* promoter methylation status (B) in response to benzo[a]pyrene (BaP) in lung A549 and H1793 cells and Jurkat T cells. Cells were treated with 0.1, 1 or 10nM BaP, with DMSO as control, every 2 days for a total of 4 days. Triplicate experiments were performed. **A:** RNA was isolated, reverse transcribed and amplified by real-time PCR. The 2-ΔΔCt method was used to calculate the relative expression level (RER) of *IL4* transcripts normalized to β-actin. **B:** Genomic DNA was isolated and bisulfite treated prior PCR amplification of *IL4* promoter region. PCR products were subcloned into pCR2.1 vectors. Four individual clones from each experiment and a total of 12 clones from each BaP concentration were sequenced. Each row represents an individual clone of the promoter. Each circle represents a CpG site within the promoter. A total of 12 CpG sites were analyzed. Open circles represent unmethylated CpGs and closed circles represent methylated CpGs. Met%: Average percent of total CpG methylation. Standard deviation was obtained from triplicate experiments and presented as error bar.* $p<0.05$ or ** $p<0.01$ compared with untreated controls.

Table S1. Participant Characteristics (n, % of column total) and Age of Mother at Delivery (median, interquartile range) by PAH Maternal Exposure Concentrations as Measured in Air (< 2.27, > or = 2.27 ng/m^3)

Participant Characteristics	Total (N=53)	PAH<2.27 (N=26)	PAH > or = 2.27 (N=27)	p-value[a]
Ethnicity				
n, % African Americans vs Dominicans	28 (53)	13 (50)	15 (56)	0.68
Newborn's gender				
N, % Male	24 (45)	11 (42)	13 (48)	0.67
ETS				
N, % Yes	18 (34)	9 (35)	9 (33)	0.92
Public Assistance				
% Yes	22 (42)	8 (31)	14 (52)	0.12
Mother's age at delivery (years) Median (IQR)	24.5 (21.2, 28.6)	24.0 (20.5, 28.3)	24.7 (23.1, 28.8)	0.84

[a] P-values testing differences between percents of specified characteristic by PAH maternal exposure levels were obtained by Pearson's chi-square test of independence. P-values testing differences between medians of age at delivery by PAH levels were obtained by quantile regression.

Table S2. Median Percent Methylation (Interquartile Range) by *IFNγ* Region and Participant Characteristics

Participant Characteristics	*IFNγ* Region 1 (n=53)	p-value[a]	*IFNγ* Region 2 (n=53)	p-value[a]
Ethnicity				
African Americans	94.1 (88.0, 97.4)	0.29	92.5 (90.9, 98.4)	0.04
Dominicans	91.2 (86.3, 99.1)		96.7 (88.9, 100)	
Newborn's gender				
Male	93.2 (81.9, 97.6)	1.0	92.5 (87.5, 100)	0.39
Female	94.1 (90.6, 98.0)		95.8 (91.7, 100)	
Public Assistance				
Yes	94.1 (88.2, 98.0)	0.65	91.7 (83.3, 97.2)	0.06
No	92.9 (85.3, 98.0)		95.8 (91.7, 100)	
ETS				
Yes	94.1 (91.8, 98.0)	0.39	94.6 (90.0, 100)	1.0
No	92.2 (85.3, 98.0)		94.4 (91.7, 100)	
Mother's Age at Delivery (years)				
< 24.5	94.1 (88.2, 98.0)	1.0	94.4 (88.9, 100)	0.44
> or = 24.5	94.1 (85.3, 98.0)		96.3 (91.7, 100)	
PAH (ng/m^3)				
< 2.27	88.7 (79.4, 94.1)	<0.01	93.1 (86.7, 96.7)	0.30
> or = 2.27	97.1 (92.9, 99.1)		96.7 (91.7, 100)	

[a] P-values test differences between region-specific medians of percent methylation for each characteristic. Results were obtained by quantile regression.

Table S3. Regression Coefficients, β (SE) for the Estimated Associations Between LnPAH and LnPercent Methylation in *IFNγ* Region 1, Adjusted for Participant Characteristics.

	**Full Model[a,b] ($R^2 = 0.260$)**		**Final Model[a,b] ($R^2 = 0.249$)**	
Independent Variable	β x 10 (SE)	p-value	β x 10 (SE)	p-value
Ethnicity (African Americans)	0.21 (0.41)	0.62	-	-
Gender (male)	-0.71 (0.41)	0.09	-0.70 (0.38)	0.07
ETS (yes)	0.13 (0.43)	0.76	-	-
Public Assist (yes)	0.18 (0.43)	0.69	-	-
LnPAH (ln ng/m^3)				
Linear	1.11 (0.70)	0.12	1.07 (0.31)	<0.01
Nonlinear Spline	-0.07 (0.65)	0.91	-	-
Age at Delivery (yr)				
Linear	-0.20 (0.12)	0.11	-0.21 (0.11)	0.08
Nonlinear Spline	0.23 (0.12)	0.07	0.23 (0.12)	0.06

[a] Full model includes all participant characteristics plus PAH. The final (reduced) model includes characteristics that were significant (p< 0.20) in the full model.

[b] In the full model for Region 1, the linear and nonlinear terms of spline lnPAH were calculated from a restricted cubic spline function which included three knots located at PAH=1.1, (10th percentile), 3.5 (PAH value where the spline function changed from increasing to decreasing), and 5.0 (90th percentile). These turning points were identified by the GAM (exploratory) graphs. Based on the LRT statistics, (i) the addition of nonlinear lnPAH in the full model did not significantly improve the model fit (p >0.20), however (ii) lnPAH remained statistically significant (p<0.20). The positivity of the regression coefficient indicated that an increase in lnPAH was associated with an increase in ln-percent methylation (β x 10= + 1.07, p<0.01). Based on the LRT, linear lnPAH was statistically significant in the reduced model. The linear trend of lnPAH in the adjusted reduced model is shown in Figure 3A.

The linear and nonlinear terms of the spline function of age at delivery in the full and reduced models were calculated from a restricted cubic spline function which included three knots located at age =19 (10th percentile), 25 (value of age at delivery where the spline function changed from decreasing to increasing), and 30.5 (90th percentile) respectively. Based on the LRT statistic, the nonlinear and linear terms of age at delivery were significant when modeled separately and together. Thus the final model included the spline function of age at delivery. In the final model, the linear and nonlinear regression coefficients β x10 of age at delivery were -0.21 (p=0.08) and +0.23 (p=0.06).

Table S4. **Regression Coefficients, β (SE) for the Estimated Associations Between LnPAH and LnPercent Methylation in *IFNγ* Region 2, Adjusted for Participant Characteristics.**

	**Full Model[a,b] (R^2 = 0.354)**		**Final Model[a,b] (R^2 = 0.348)**	
Independent Variable	β x 10 (SE)	p-value	β x 10 (SE)	p-value
Ethnicity (African Americans)	-0.07 (0.20)	0.72	-	-
Gender (male)	-0.10 (0.20)	0.64	-	-
ETS (yes)	0.29 (0.21)	0.18	0.29 (0.21)	0.17
Public Assist (yes)	-0.73 (0.21)	<0.01	-0.73 (0.21)	<0.01
LnPAH (ln ng/m^3)				
Linear	1.13 (0.33)	<0.01	1.15 (0.31)	<0.001
Nonlinear Spline	-0.55 (0.30)	0.07	-0.57 (0.28)	0.04
Age at Delivery (yr)				
Linear	-0.15 (0.11)	0.19	-0.14	0.22
Nonlinear Spline 1	0.84 (0.43)	0.06	0.82	0.07
Nonlinear Spline 2	-1.57 (0.78)	0.05	-1.53	0.06

[a] Full model includes all participant characteristics plus PAH. The final (reduced) model includes characteristics that were significant (p< 0.20) in the full model.

[b] The spline of lnPAH in the full and reduced models included three knots located at PAH=1.1, (10th percentile), 3.5 (PAH value where the spline function changed from increasing to decreasing), and 5.0 (90th percentile). Based on the LRT statistics, the nonlinear *and* linear terms of lnPAH were significant when modeled separately and together in the adjusted full and final models. .In the final model, the linear and nonlinear coefficients, β x10, of lnPAH were +1.15 (p<0.001) and -0.57 (p=0.04) respectively. Figure 3B shows that percent methylation increased as PAH increased until PAH reached approximately 3.5 ng/m^3, and then the curve decreased, as suggested by the exploratory (GAM) graphs..

The spline function of age at delivery in the full *and* reduced models included four knots located at age =18.5 (10th percentile), 21.8 (age value where the first change in the slope of the spline function occurred-decreasing to increasing), 29.1 (age value where the second change occurred - increasing to decreasing), and 30.5 (90th percentile). In the final model, the linear and two nonlinear coefficients, (β x10), of age at delivery were -0.14 (p=0.22), +0.82 (p=0.07) and -1.53 (p=0.06) , respectively. Based on LRT statistics, the two nonlinear and the linear terms of age at delivery were significant when modeled separately and together.

Supplemental Material, References

Reddy MV, Randerath K. 1986. Nuclease P1-mediated enhancement of sensitivity of ^{32}P-postlabeling test for structurally diverse DNA adducts. Carcinogenesis 7(9): 1543-1551.

Zavon MR, Hoegg U, Bingham E. 1973. Benzidine exposure as a cause of bladder tumors. Arch Environ Health. 27(1):1-7.
